# Photon-Counting Underwater Optical Wireless Communication for Reliable Video Transmission Using Joint Source-Channel Coding Based on Distributed Compressive Sensing

**DOI:** 10.3390/s19051042

**Published:** 2019-03-01

**Authors:** Zhu Hong, Qiurong Yan, Zihang Li, Ting Zhan, Yuhao Wang

**Affiliations:** 1 School of Information Engineering, Nanchang University, Nanchang 330031, China; hongzhu910@126.com (Z.H.); lizihang@email.ncu.edu.cn (Z.L.); zhanting322ncu@163.com (T.Z.); wangyuhao@ncu.edu.cn (Y.W.); 2 State Key Laboratory of Transient Optics and Photonics, Xi’an Institute of Optics and Precision Mechanics, Chinese Academy of Sciences, Xi’an 710119, China

**Keywords:** photon-counting, underwater optical wireless communication, distributed compressive video sensing

## Abstract

To achieve long-distance underwater optical wireless communication, a single photon detector with single photon limit sensitivity is used to detect the optical signal at the receiver. The communication signal is extracted from the discrete single photon pulses output from the detector. Due to fluctuation of photon flux and quantum efficiency of photon detection, long-distance underwater optical wireless communication has the characteristics that the link is easily interrupted, the bit error rate is high, and the burst error is large. To achieve reliable video transmission, a joint source-channel coding scheme based on residual distributed compressive video sensing is proposed for the underwater photon counting communication system. Signal extraction from single photon pulses, data frame and data verification are specifically designed. This scheme greatly reduces the amount of data at the transmitter, transfers the computational complexity to the decoder in receiver, and enhances anti-channel error ability. The experimental results show that, when the baud rate was 100 kbps and the average number of photon pulses per bit was 20, the bit error rate (BER) was 0.0421 and video frame could still be restored clearly.

## 1. Introduction

Underwater optical wireless communication (UOWC) plays an important role in military, environmental detection, marine exploration and disaster prevention, and has attracted more and more attention in recent years [1,2,3]. The communication distance is limited due to serious absorption and scattering of water [4]. To achieve long-distance underwater communication, a single photon detector with single photon limit sensitivity is used to detect very weak light signal at the receiver [5,6,7,8,9,10,11]. The communication signals are extracted from the discrete single photon pulses that output from single photon detector.

Video communication is an important application aspect of underwater optical wireless communication because of its vivid and picturesque characteristic. Due to fluctuation of photon flux and quantum efficiency of photon detection, long-distance photon counting underwater optical wireless communication has the characteristics that the link is easily interrupted, the bit error rate is high, and the burst error is large. It is necessary to improve the transmission reliability through error correction coding. Traditional video compression standards, such as MPEG or H.26X, use a hybrid coding method combining predictive coding and transform coding to compress video sequences, which make video frames depend heavily on each other [12,13]. Thus, if a data frame were lost, other data frames would not be decoded. In a photon-counting underwater optical wireless communication system, the signal in a time slot is a discrete single-photon pulse sequence, and even there no photon is detected in many time slots, causing a lot of symbol deletion and packet loss. Therefore, the traditional video compression standards and coding have great difficulties to restore video [14,15].

In 2006, Tao et al. proposed a compressed sensing (CS) theory, which broke through the limitation of traditional Nyquist sampling theorem [16,17]. According to the CS theory, signal can be sampled and compressed at the same time; as a result, it is widely used in the field of video compression [18,19,20,21,22,23]. In 2008, Stanković et al. [18] firstly applied compressed sensing theory to video coding, and proposed compressive video sampling (CVS). On the other hand, this paper proposes to divide video frames into key frames and non-key frames, where key frames use traditional video coding, while non-key frames use compressed sensing coding. The experimental results show that, on conditions of a better reconstruction, the method saves nearly half of the video collection. In 2009, Kang et al. proposed distributed compressive video sensing (DCVS) based on inter-frame correlation [19,20]. The encoding end allocates different measurement rates to key frames and non-key frames to reduce the amount of data transmitted, and each frame is compressed by CS measurement. The decoding end uses joint decoding to reconstruct, while the amount of data at the transmitting end is reduced and the complexity is transmitted to the decoding end. Chen et al. [21] investigated dynamic measurement rate allocation in block-based DCVS, which can adaptively adjust measurement rates by estimating the sparsity of each block via feedback information. Chen et al. [22] proposed residual distributed compressive video sensing based on double side information (RDCVS-DSI). In RDCVS-DSI, by taking advantage of the frequency domain characteristics of the image and the correlation between consecutive frames, the low-quality video frames are regarded as the first side information in the encoding process, and the second side information is generated from the motion estimation of non-key frames. Performance analysis and simulation results show that the RDCVS-DSI model can reconstruct the high-fidelity video sequence with lower complexity.

In this paper, to realize reliable video transmission of long-distance optical wireless communication, a joint source-channel coding scheme based on residual distributed compressive video sensing is proposed for the underwater photon counting communication system with high bit error rate. Signal extraction from single photon pulses, data frame and data verification are specifically designed. Experimental results show that this scheme greatly reduced the amount of data at the transmitter, transfered the coding complexity to the receiver, and enhanced the anti-channel error ability.

## 2. Joint Source-Channel Coding Scheme Based on Residual Distributed Compressive Video Sensing

According to the CS theory, accurate reconstruction of a signal can be obtained by sampling the signal in a small amount, when the signal is sparse or the signal can be sparse under certain conditions. Compressed sensing theory can be abstracted into the mathematical expression y = Φx, where *x* is sparse signal of *N* dimensions, *Φ* is measurement matrix with dimension *M* × *N*, and *y* is measured value. If the signal *x* has a basic base in a certain domain, e.g., *x* = *Ψα*, where *α* is a sparse signal and *Ψ* is a sparse matrix with dimension *N* × *N*, the mathematical expression becomes *y* = *ΦΨα*. In reconstruction process, the value of *α* is obtained by solving the underdetermined equations, and the signal *x* is obtained by *α*. The algorithm is usually solved by Orthogonal Matching Pursuit (OMP) [24], Total variation Augmented Lagrangian Alternating Direction Algorithm (TVAL3) [25], etc.

**Figure 1:**
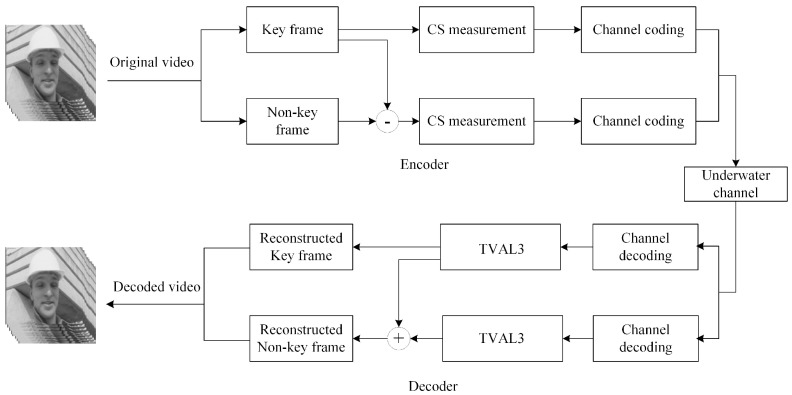
Joint source-channel coding scheme based on residual distributed compressive video sensing.

Joint source-channel coding scheme based on residual distributed compressive video sensing is shown in Figure 1. A video sequence consists of several GOPs (group of pictures), where a GOP consists of a key frame and non-key frame; each frame can be seen as a sparse signal of *N* dimensions [20]. To ensure the reconstruction performance and reduce the amount of data, the key frame adopts a higher measurement rate for the compressed sensing measurement. Besides, an extremely sparse residual matrix is obtained by subtracting key frames from non-key frames, and a lower measurement rate for the compressed sensing measurement is adopted. Suppose that *Φ_k_* is measurement matrix of key frame with dimension *M_k_* × *N*, *Φ_cs_* is measurement matrix of residual matrix with dimension *M_k_* × *N*, the measurement rate of key frames is *r_k_* = *M_k_/N*, and the measurement rate of residuals matrix is *r_cs_* = *M_cs_/N*. Assuming that the number of key frames is *n_k_*, the number of residuals matrix is *n_cs_*, respectively. Then, the average measurement rate (AMR) is:

(1) $$r_{ave}=\frac{\left(r_{k}\times n_{k}+r_{cs}\times n_{cs}\right)}{\left(n_{k}+n_{cs}\right)}$$

**Figure 2:**
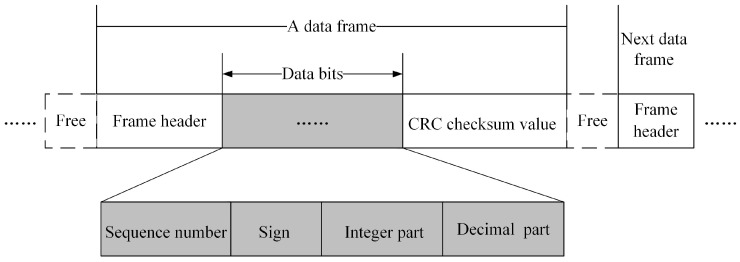
Data frame format.

Each measured value is converted into a data frame for transmission in the underwater channel, and the data frame format is shown in Figure 2. The data frame consists of the frame header “FF FF FF FF”, the sequence number of the measured value in the matrix, the positive and negative sign, integer and decimal part of the measured value, and CRC (Cyclic Redundancy Check) checksum value. In this way, a measured value is packed. Transmitter and receiver follow this predetermined data format for transmission and reception.

Receiver decodes the received packets and restores the measured value. According to the CRC, the correct measured value is placed in the measurement matrix by the serial number, and the wrong measured value is discarded (assignment 0). The measurement matrix is generated by setting the same random number seed as the sender; it does not need to be transmitted. The measured value matrix and the corresponding measurement matrix can be used to reconstruct the image frame by compression sensing algorithm. The key frames are reconstructed directly. The non-key frames are obtained by summing the reconstructed residual matrix and the key frames. Finally, the reconstructed video frame is restored to the video.

Light propagation in water suffers from attenuation through both absorption and scattering, which may cause data loss or bit error. The measured values determined by the distributed compressive video sensing theory are of equal importance. Thus, the reconstructed picture quality at the decoder depends only on the number and correctness of the received measured values, i.e., the impact of any data loss is apportioned to each frame of video. Therefore, the loss of measured values has little impact on the reconstruction quality of the image.

## 3. System Principle and Realization

**Figure 3:**
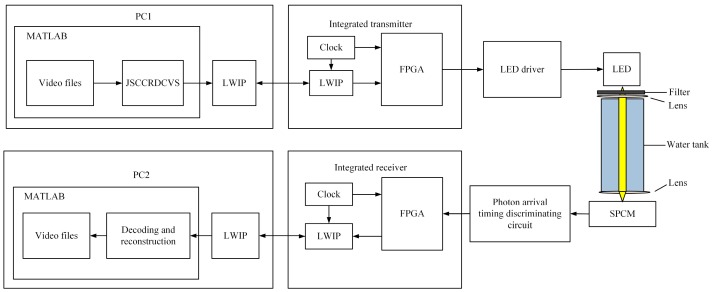
The structure diagram of system. LED, led lamp; SPCM, single photon counter module; FPGA, field programmable gate array; JSCCRDCVS, joint source and channel coding scheme based on residual distributed compressive video sensing; LWIP, a lightweight TCP/IP stack; PC, personal computer.

The structure diagram of system is shown in Figure 3. The whole system is mainly composed of two parts: the transmitter and the receiver. The transmitter uses MATLAB to code video frame sequence based on the distributed compressive sensing theory. The encoded data are transmitted to FPGA (DIGILENT, ZYNQ-7000) via LWIP protocol, FPGA of the transmitter performs OOK (On-Off Keying) modulation on LED (CREE, Q5) through the driving circuit. The receiver transforms the discrete photon pulses into continuous bit information by utilizing the time interval of photons arrival, and then sends the continuous bit information to MATLAB for decoding via LWIP protocol.

**Figure 4:**
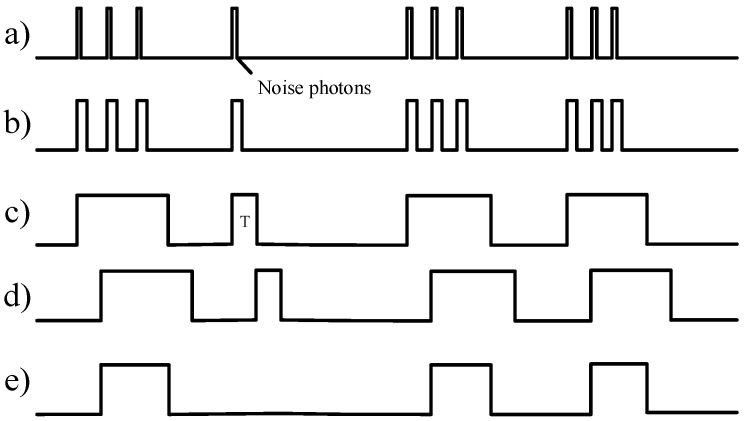
Timing of signal extraction: (**a**) single-photon pulse signal output by the detector; (**b**) stretched single-photon pulse signal; (**c**) trailing demodulated signal; (**d**) shift register output signal; and (**e**) demodulated signal.

The principles of restoring the signal is shown in Figure 4. The timing of signal extraction method has the following steps: (1) To facilitate sampling with a 50 M clock, the single-photon pulse signal output by the detector is stretched. The width of stretched single-photon pulse signal is greater than 40 ns. (2) The FPGA sets a threshold “T” whose type is time interval. The trailing demodulator outputs a signal “1” when the stretched single-photon pulse signal is detected, and if FPGA can detect the next stretched signal within “T”, the trailing demodulator keeps on outputting the signal “1” until the stretched signal does not arrive during the “T”. Then, the trailing demodulator outputs signal “0”. (3) The FPGA sends the trailing demodulation signal to the shift register whose width of the right shift is “T”. Moreover, “T” is also the redundant length of the tail demodulation signal. Then, the shift registers output signal and the trailing demodulation signal are logically AND to obtain a true demodulated signal. (4) The above operation is repeated.

**Figure 5:**
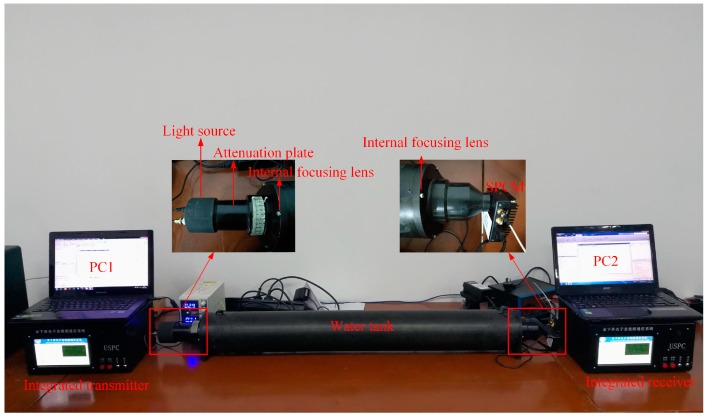
Photon-counting underwater optical wireless communication system.

Figure 5 draws a practical picture of photon-counting underwater optical wireless communication system. PC1 uses the MATLAB platform to encode the video based on the distributed compressive sensing theory. Then, the measured values are converted into data frames, which are loaded into the integrated transmitter via the network port. In the system, OOK modulation method is utilized and the LED driving circuit is applied to transform the encoded binary bit stream into optical signal. The length of cylindrical water tank is 150 cm, which is filled with still pure water. After the optical signal passing through the water tank to the single photon detector, the single photon detector receives the extremely weak optical signal and outputs the discrete single photon pulse sequence. A specially designed demodulator extracts data from discrete single photon pulse sequences at the integrated receiver. The data arrive at PC2 via the network port. PC2 uses the MATLAB platform to decode the received data stream and restore the measured value. Finally, the video frames are reconstructed and the video is restored.

Comparing the reconstructed video frames with the original images, the image quality assessment is usually divided into two categories: subjective quality evaluation and objective quality evaluation. Subjective quality evaluation depends on the human eye’s intuitive perception of the image [26]. Objective quality evaluation depends on formula, such as mean-square error (MSE), Peak Signal to Noise Ratio (PSNR) and so on. The expression of *MSE* is as follows:

(2) $$MSE=\frac{\sum_{i=1}^{M}\sum_{j=1}^{M}{\left(f_{ij}-f_{ij}\prime \right)}^{2}}{M\times N}$$

Where *f_ij_* and *f_ij_’* represent the original image and the restored image, respectively, and 1 ≤ *i* ≤ *M*, 1 ≤ *j* ≤ *M*. The expression of *PSNR* is as follows:

(3) $$PSNR=10\mathrm{lg}\frac{255\times 255}{MSE}$$

**Figure 6:**
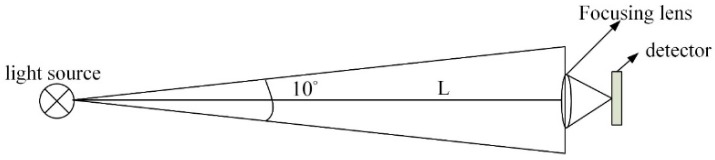
Optical signal transmission model of underwater optical communication.

Figure 6 shows the optical signal transmission model of our underwater optical communication system. Emission power rate of LED is 1.1 W, and the emission angle of the collimator is 5°. The formula of receiving power for underwater optical communication is as follows [27]:

(4) $$P_{r}=P_{t}\cdot \exp(-c\cdot L)$$

where *c* is the attenuation coefficient, *P_t_* is transmitting power, and *L* is the longest communication distance.

## 4. Experimental Results and Discussion

**Figure 7:**
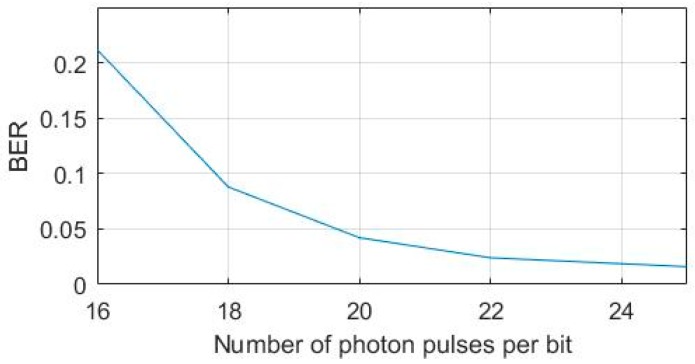
BER at different average number of photon pulses per bit.

To verify the effectiveness of distributed compressive video sensing coding method in photon-counting underwater wireless optical communication system, we used the standard test video sequences “foreman” and “coastguard” (frame size: 128 × 128) as experimental object, with GOP size = 3. The first frame was a key frame, the second frame and the third frame are non-key frames, and the reconstruction algorithm used TVAL3. As a result of the limited length of the water tank, longer distance communication was simulated by attenuating the power of the LED, and the attenuation in the direction of communication was mainly caused by scattering and absorption. Since the communication rate was low and the communication distance was short, scattering was considered the only cause of attenuation, while other impacts caused by scattering were very small and therefore ignored. Therefore, we did not consider the effect of scattering on this experiment, even though it might be somewhat beneficial when some photons are scattered back to the receiver. Instead, we considered the transmitted power having the main effect on the number of received photons. In addition, we simulated the attenuation of light transmission in water by changing the voltage of the LED lamp. Figure 7 shows the BER at different number of photon pulses per bit calculated at the detector, and the baud rate of 100 kbps. The received optical power evaluation index used in this experiment was the number of single photon pulses output by the detector. It can be seen in the figure that the weaker was the attenuation process, i.e., the more photon pulses per bit, the lower was the bit error rate.

**Figure 8:**
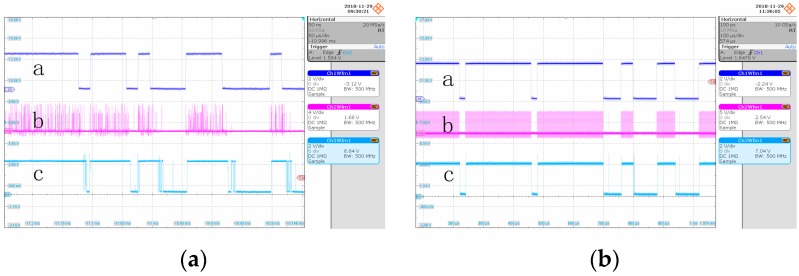
Waveform of underwater photon-counting communication at different BER: (**a**) BER = 0.1530; and (**b**) BER = 0.0210.

Figure 8 shows the waveforms of underwater photon-counting communication at different BER, where a is the modulation signal, b is the single photon pulse signal output by the detector, and c is the demodulation signal. It can be seen in the figure that, when the bit error rate was small, the demodulation signal waveform did not have pitting distortion and tailing phenomenon. When the bit error rate was high, the discrete single photon pulse signal became sparse, the number of photon pulses in the high level decreased, and the demodulation signal part presented pitting distortion.

**Figure 9:**
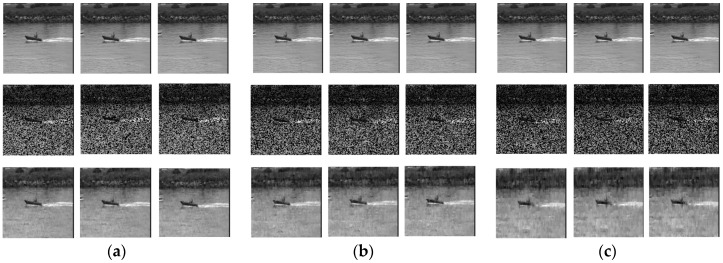
Comparison of the first three frames of “coastguard “at different BER: (**a**) BER = 0.0062; (**b**) BER = 0.0081; and (**c**) BER = 0.0107. The first line of pictures are the original video frames, the second line of pictures are the result of direct transmission of video frames, and the third line of pictures is the result of transmission of video frames after distributed compressive video sensing coding.

Firstly, we used the standard test video sequences “coastguard” as experimental object. It does not need distributed compressive video sensing coding operation. Video frames data were directly transmitted through the underwater photon-counting communication system after channel coding. Then, video frames were measured by distributed compressive video sensing coding, and the measured values were transmitted through the underwater communication system after channel coding. Finally, video frames were restored by decoding at the receiver. The experimental results show the comparison of the first three frames of “coastguard” at different BER. In Figure 9, the first line of pictures are the original video frames, the second line of pictures are the result of direct transmission of video frames, and the third line of pictures are the result of transmission of video frames after distributed compressive video sensing coding. When the bit error rate was high, directly transmitted video frames data could not receive clear pictures.

**Figure 10:**
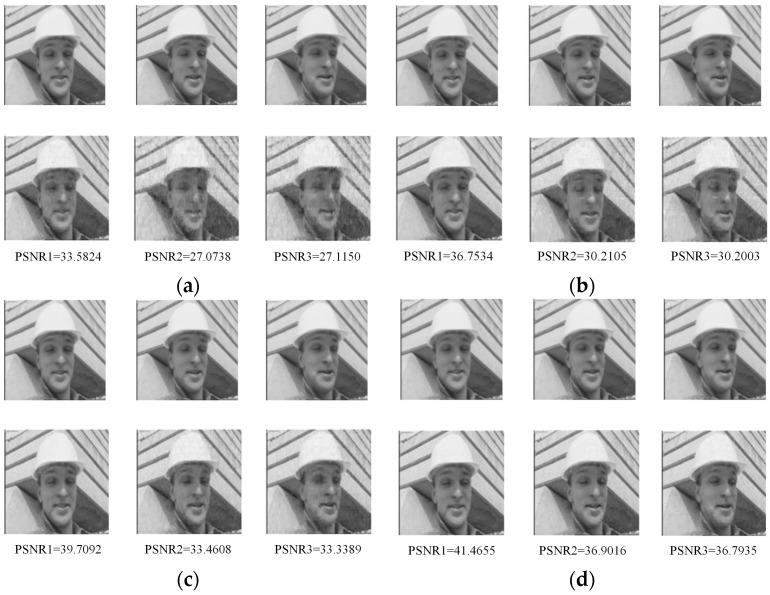
Comparison of the first three frames of “foreman” at different AMR: (**a**) AMR = 0.4667; (**b**) AMR = 0.5667; (**c**) AMR = 0.6667; and (**d**) AMR = 0.7667.

To evaluate the image reconstruction performance subjectively at different average measurement rate, the comparison of the first three frames of the “foreman” is shown in Figure 10 when the average measurement rates were 0.4667, 0.5667, 0.6667 and 0.7667. As can be seen in Figure 10, the key frame was the first frame, and the other frames were non-key frames. The measurement rate of the key frame was higher, the reconstructed image was clearer, and the PSNR value was larger.

**Figure 11:**
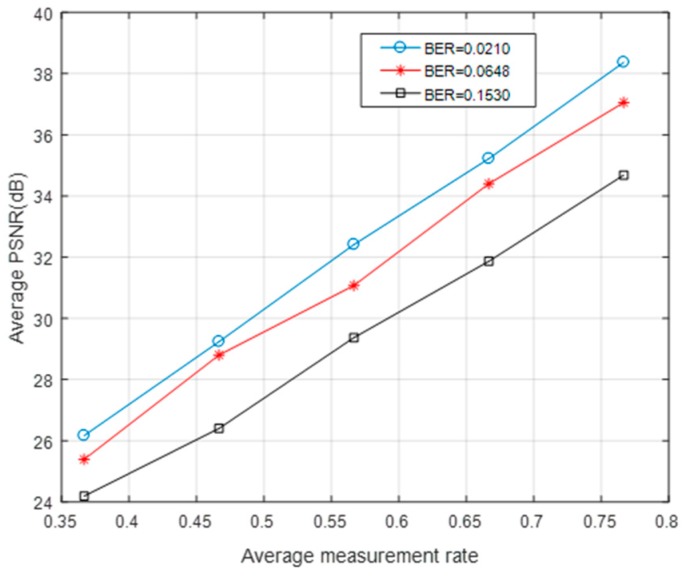
The AMR-PSNR performances for the “foreman”.

**Figure 12:**
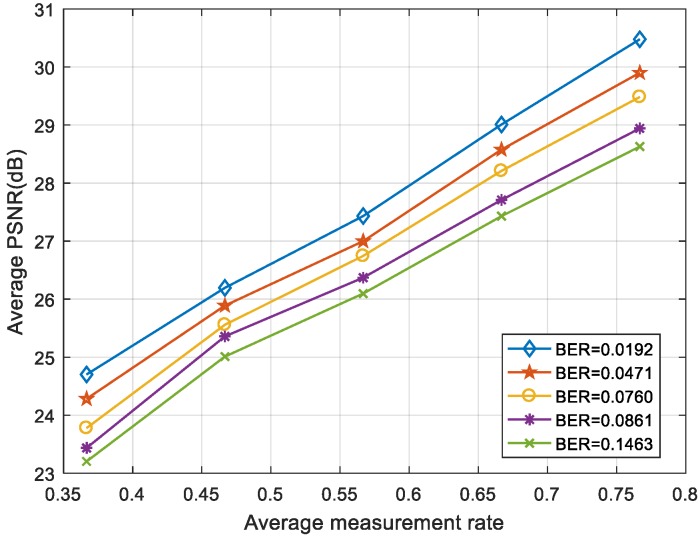
The AMR-PSNR performances for the “coastguard”.

As can be seen in Figure 11 and Figure 12, the “foreman” and “coastguard” were reconstructed under different BER. In the following figures, the abscissa is the AMR and the ordinate represents the average PSNR value of the reconstructed image. Experimental results demonstrate that the PSNR increases with average measurement rate.

The optical signal became extremely weak when it passed through the underwater channel to the single photon detector. Since the communication distance was short and the rate was low, very few photons belonging to the slot of symbol “1” traverses to the slot of symbol “0” due to scattering. The bit error we observed was mainly because the number of photons in the time slot of the symbol “1” was not enough, causing the symbol “1” to be misjudged as “0”. It was somewhat advantageous to identify the symbol “1” if the scattered photons run into the time slot of the symbol “1”. The time slot of the symbol “0” had very few scattered photons. However, the distance between the dark count and the background noise photon was much larger than the threshold we set, thus the symbol “0” was not misjudged as “1”.

**Figure 13:**
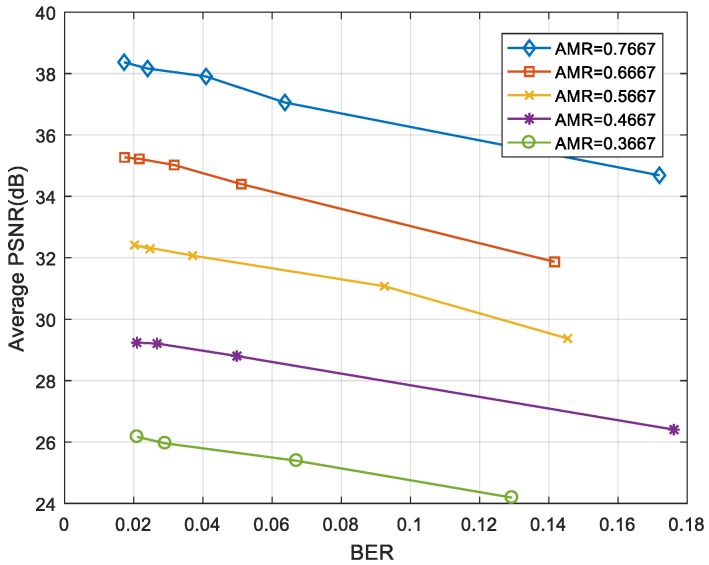
BER-PSNR performances for the “foreman”.

**Figure 14:**
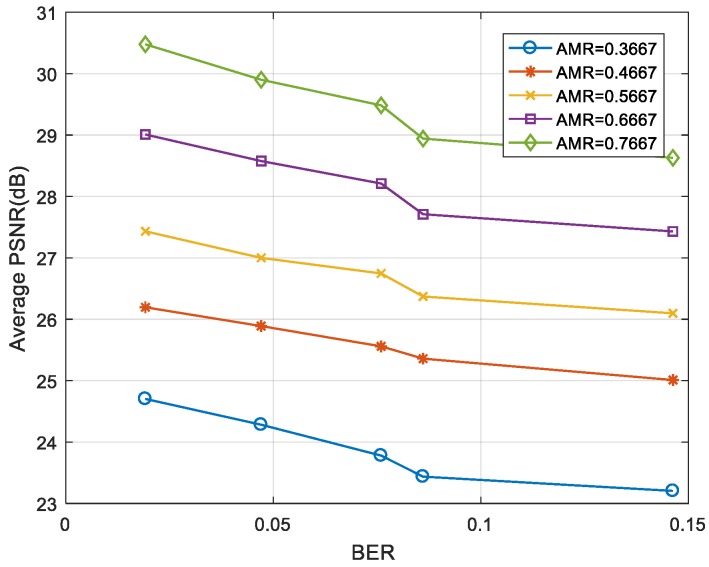
BER-PSNR performances for the “coastguard”.

Figure 13 and Figure 14 show the average PSNR of the reconstructed video sequences at different BER under five average measurement rates. The results show that the larger was the BER, the smaller was the PSNR. Even when the BER was ≥0.1, the video frame could still be restored clearly as long as the measurement rate was large enough.

The wavelength in our experiment system was 500 nm. The quantum efficiency of single photon detector was *η* = 35%. Effective photosensitive area was a circle of diameter *∅* = 500 μm. To increase the communication distance, a focusing lens with a diameter of 10 cm was placed before the single photon detector. According to the experiments, when the baud rate was 100 kbps and the average number of photon pulses per bit was 20, the bit error rate was 0.0421, and the video frame could still be restored clearly. We calculated the optical power corresponding to 20 photon pulses per bit as $1.7\times 10^{-12}$ W, according to Equation (4). As shown in Table 1, we calculated the theoretical longest communication distance of three water types. The attenuation coefficients of three water types were taken from the work done by Petzold [28]. Farther communication distance could be achieved by increasing LED power or using multiple LEDs.

## 5. Conclusions

In this study, a photon-counting underwater optical wireless communication system was built. To achieve reliable video transmission at high bit error rate, we proposed and testified a joint source-channel coding scheme based on residual distributed compressive video sensing, and the transmitted information was specially designed in data frame. In the experiment, the influence of AMR and BER on the quality of reconstructed video frames was analyzed, and the results verify that, if the measurement rate was large enough, the video frames could be restored clearly in the high bit error rate communication environment. This scheme overcomes the traditional video coding shortcomings, such as large computational complexity, limited storage capacity and high coding complexity. It transfers the coding complexity to the decoder, and reduces the sampled data and the requirement of hardware caching. The experimental results show that, when the baud rate was 100 kbps and the average number of photon pulses per bit was 20, the bit error rate was 0.0421 and video frame could still be restored clearly.

## Figures and Tables

**Table 1:** Theoretical longest communication distance for three water types.

Water Type	c (m^−1^)	L (m)
Harbor	2.19	8.16
Coastal	0.40	40.66
Clear	0.15	102.27
